# Supplementary material for: The association between caesarean section delivery and obesity at age 17 years. Evidence from a longitudinal cohort study in the United Kingdom
Source: PLoS One. 2024 May 31;19(5):e0301684. doi: 10.1371/journal.pone.0301684 (PMC11142666; doi:10.1371/journal.pone.0301684)
Supplement: S1 Table — (PDF) [file pone.0301684.s001.pdf]

Supporting information

S1 Table: Maternal characteristics and pregnancy complications characteristics related to mode of delivery among Millennium Cohort Study participants

	Normal VD n (%)	Assisted VD n (%)	Planned CS n (%)	Emergency CS n (%)	Overall n (%)
	6,090 (68.6)	867 (9.8)	808 (9.1)	1,115 (12.6)	8,880 (100.0)
**Maternal Characteristics**					
Maternal Age (years)					
Median (IQR)	29 (25-33)	29 (25-33)	32 (28-35)	30 (26-34)	29 (25-33)
Ethnicity					
White	4,905 (80.5)	762 (87.9)	675 (83.5)	889 (79.7)	7,231 (81.4)
Mixed	173 (2.8)	25 (2.9)	18 (2.2)	36 (3.2)	252 (2.8)
Indian	177 (2.9)	22 (2.5)	17 (2.1)	38 (3.4)	254 (2.9)
Pakistani/Bangladeshi	525 (8.6)	39 (4.5)	59 (7.3)	65 (5.8)	688 (7.8)
Black/Black British	201 (3.3)	13 (1.5)	23 (2.9)	61 (5.5)	298 (3.4)
Other/Missing	96 (1.6)	6 (0.7)	14 (1.7)	26 (2.3)	142 (1.6)
Missing	13 (0.2)	0 (0.0)	2 (0.3)	0 (0.0)	15 (0.2)
Highest Maternal Education Achieved					
Less than O level	,1016 (16.7)	70 (8.1)	104 (12.9)	130 (11.7)	1,320 (14.9)
O level	2,517 (41.3)	333 (38.4)	318 (39.4)	425 (38.1)	3,593 (40.5)
A level	629 (10.3)	115 (13.3)	71 (8.8)	121 (10.9)	936 (10.5)
Diploma or above	629 (10.3)	115 (13.3)	71 (8.8)	121 (10.9)	936 (10.5)
Other/Missing	204 (3.4)	21 (2.4)	24 (3.0)	37 (3.3)	286 (3.2)
Household Income					
Lowest quintile	1,324 (21.7)	121 (14.0)	114 (14.1)	155 (13.9)	1,714 (19.3)
Second quintile	1,353 (22.2)	124 (14.3)	153 (18.9)	192 (17.2)	1,822 (20.5)
Third quintile	1,140 (18.7)	177 (20.4)	168 (20.8)	214 (19.2)	1,699 (19.1)
Fourth quintile	1,158 (19.0)	210 (24.2)	183 (22.7)	251 (22.5)	1,802 (20.3)
Highest quintile	1,096 (18.0)	234 (27.0)	189 (23.4)	303 (27.2)	1,822 (20.5)
Missing	19 (0.3)	1 (0.1)	1 (0.1)	0 (0.0)	21 (0.2)
Marital Status					

	**Normal VD n (%)**	**Assisted VD n (%)**	**Planned CS n (%)**	**Emergency CS n (%)**	**Overall n (%)**
Legally separated	191 (3.1)	13 (1.5)	17 (2.1)	22 (2.0)	243 (2.7)
1st Marriage	3,648 (59.9)	535 (61.7)	570 (70.5)	704 (63.1)	5,457 (61.5)
2nd Marriage	252 (4.1)	29 (3.3)	53 (6.6)	60 (5.4)	394 (4.4)
Never married	1,737 (28.5)	258 (29.8)	130 (16.1)	291 (26.1)	2,416 (27.2)
Divorced	249 (4.1)	31 (3.6)	36 (4.5)	37 (3.3)	353 (4.0)
Widowed	12 (0.2)	1 (0.1)	2 (0.3)	1 (0.1)	16 (0.2)
Fertility assistance					
Yes	117 (1.9)	29 (3.3)	32 (4.0)	55 (4.9)	233 (2.6)
No	3,308 (54.3)	530 (61.1)	492 (60.9)	653 (58.6)	4,983 (56.1)
Not applicable	2,665 (43.8)	308 (35.5)	284 (35.1)	407 (36.5)	3,664 (41.3)
Maternal Smoking During Pregnancy					
Non-smoker	4,222 (69.3)	608 (70.1)	612 (75.7)	792 (71.0)	6,234 (70.2)
Quit during pregnancy	666 (10.9)	127 (14.7)	91 (11.3)	140 (12.6)	1,024 (11.5)
Smoked during pregnancy	1,200 (19.7)	131 (15.1)	105 (13.0)	183 (16.4)	1,619 (18.2)
Missing	2 (0.03)	1 (0.1)	0 (0.0)	0 (0.0)	3 (0.03)
Alcohol Consumption During Pregnancy					
No	4,187 (68.8)	585 (67.5)	561 (69.4)	792 (71.0)	6,125 (69.0)
Yes	1,901 (31.2)	282 (32.5)	247 (30.6)	323 (29.0)	2,753 (31.0)
Missing	2 (0.03)	0 (0.0)	0 (0.0)	0 (0.0)	2 (0.02)
**Pregnancy Complications**					
Preterm (<37weeks)					
Yes	303 (5.0)	40 (4.6)	54 (6.7)	193 (17.3)	590 (6.6)
No	5,726 (94.0)	817 (94.2)	745 (92.2)	913 (81.9)	8,201 (92.4)
Missing	61 (1.0)	10 (1.2)	9 (1.1)	9 (0.8)	89 (1.0)
Macrosomia (>4000g)					
Yes	726 (11.9)	109 (12.6)	106 (13.1)	195 (17.5)	1,136 (12.8)
No	5,364 (88.1)	758 (87.4)	702 (86.9)	920 (82.5)	7,744 (87.2)
Small for Gestational Age					
Yes	566 (9.3)	72 (8.3)	53 (6.5)	133 (11.9)	824 (9.3)
No	5,458 (89.6)	785 (90.5)	743 (92.0)	973 (87.3)	7,959 (89.6)
Missing	66 (1.1)	10 (1.2)	12 (1.5)	9 (0.8)	66 (1.1)
Hypertensive Disorder of Pregnancy					

	**Normal VD n (%)**	**Assisted VD n (%)**	**Planned CS n (%)**	**Emergency CS n (%)**	**Overall n (%)**
Yes	336 (5.5)	77 (8.9)	71 (8.8)	167 (15.0)	651 (7.3)
No	5,754 (94.5)	790 (91.1)	737 (91.2)	948 (85.0)	8,229 (92.7)

Abbreviations: VD = vaginal delivery, CS = Caesarean section, IQR = interquartile range
