# Supplementary material for: The association between caesarean section delivery and obesity at age 17 years. Evidence from a longitudinal cohort study in the United Kingdom
Source: PLoS One. 2024 May 31;19(5):e0301684. doi: 10.1371/journal.pone.0301684 (PMC11142666; doi:10.1371/journal.pone.0301684)
Supplement: S2 Table — (PDF) [file pone.0301684.s002.pdf]

S2 Table: Logistic Regression models examining the association between Mode of Delivery and obese/overweight BMI at 17 years adjusting for maternal pre-pregnancy BMI and diabetes separately.

	Model 2[a]		Model 2[a] + pre-pregnancy BMI		Model 2[a] + pre-pregnancy diabetes		Model 4[b]	
**Mode of Delivery**	**OR (95% CI)**	**p-value**	**OR (95% CI)**	**p-value**	**OR (95% CI)**	**p-value**	**OR (95% CI)**	**p-value**
Normal VD	ref		ref		ref		ref	
**Overweight**								
Assisted VD	1.02 (0.84 - 1.25)	0.814	1.02 (0.84-1.24)	0.848	1.02 (0.84 – 1.25)	0.821	1.02 (0.84-1.24)	0.849
Planned CS	1.15 (0.95-1.4)	0.154	1.05 (0.86 – 1.28)	0.608	1.14 (0.94 – 1.38)	0.193	1.05 (0.86-1.28)	0.633
Emergency CS	1.15 (0.97-1.37)	0.118	1.05 (0.88 – 1.25)	0.593	1.14 (0.96 – 1.36)	0.130	1.05 (0.88-1.25)	0.599
**Obese**								
Assisted VD	0.89 (0.68-1.17)	0.392	0.88 (0.66-1.16)	0.369	0.88 (0.67- 1.16)	0.364	0.88 (0.66-1.16)	0.356
Planned CS	1.15 (0.89-1.49)	0.270	0.97 (0.74-1.26)	0.805	1.11 (0.86 - 1.44)	0.430	0.95 (0.72-1.24)	0.688
Emergency CS	1.42 (1.15-1.76)	**0.001**	1.19 (0.96 – 1.48)	0.121	1.40 (1.13 -1.73)	**0.002**	1.18 (0.95-1.47)	0.138

Abbreviations: VD=vaginal delivery, CS = Caesarean section, CI = confidence interval, OR = odds ratio, ref = reference

[a]Adjusted for maternal and cohort member characteristics: maternal age/education/smoking/alcohol/ marital status/income/ethnicity, fertility assistance, infant sex, parity.

[b]Adjusted for maternal and cohort member characteristics as above and pre-pregnancy maternal BMI and maternal diabetes
