# Supplementary material for: The association between caesarean section delivery and obesity at age 17 years. Evidence from a longitudinal cohort study in the United Kingdom
Source: PLoS One. 2024 May 31;19(5):e0301684. doi: 10.1371/journal.pone.0301684 (PMC11142666; doi:10.1371/journal.pone.0301684)
Supplement: S3 Table — (PDF) [file pone.0301684.s003.pdf]

S3 Table. Multinomial Logistic Regression examining the association between Mode of Delivery and BMI category at age 17 years among Millennium Cohort Study participants

	Underweight		Overweight		Obese	
	RRR (95% CI)	p-value	RRR (95% CI)	p-value	RRR (95% CI)	p-value
**Mode of Delivery**						
**Normal VD**	Ref		Ref		Ref	
**Assisted VD**	1.07 (0.84–1.37)	0.566	1.01 (0.83-1.23)	0.919	0.89 (0.68-1.17)	0.406
**Planned CS**	1.07 (0.83-1.38)	0.595	1.06 (0.87-1.29)	0.569	0.96 (0.75-1.25)	0.776
**Emergency CS**	1.01 (.0.80-1.27)	0.942	1.05 (0.88-1.25)	0.600	1.19 (0.96-1.47)	0.124

Abbreviations: VD=vaginal delivery, CS = Caesarean section, CI = confidence interval, OR = odds ratio, ref = reference
Adjusted for maternal and cohort member characteristics, maternal health characteristics, and pregnancy complications as per Model 5
