# Supplementary material for: The association between caesarean section delivery and obesity at age 17 years. Evidence from a longitudinal cohort study in the United Kingdom
Source: PLoS One. 2024 May 31;19(5):e0301684. doi: 10.1371/journal.pone.0301684 (PMC11142666; doi:10.1371/journal.pone.0301684)
Supplement: S4 Table — (PDF) [file pone.0301684.s004.pdf]

S4 Table: Model 5 Adjusted Logistic Regression examining the association between Mode of Delivery including induction of labour and BMI category at age 17 years excluding induced planned CS

	**Underweight**		**Overweight**		**Obese**	
	**OR (95% CI)**	**p-value**	**OR (95% CI)**	**p-value**	**OR (95% CI)**	**p-value**
**Mode of Delivery & Induction status**						
**Normal VD**						
Not induced	Ref		Ref		Ref	
Induced	0.96 (0.78-1.16)	0.650	1.16(1.00-1.65)	**0.048**	1.06(0.87-1.29)	0.566
**Assisted VD**						
Not induced	0.91(0.67-1.26)	0.598	1.04(0.80-1.34)	0.775	0.78(0.53-1.14)	0.205
Induced	1.23(0.86-1.77)	0.257	1.10(0.82-1.48)	0.518	0.99(0.66-1.47)	0.953
**Planned CS**						
Not induced	1.05(0.80-1.39)	0.719	1.12(0.90-1.39)	0.297	0.96(0.72-1.29)	0.801
**Emergency CS**						
Not induced	1.14(0.85-1.53)	0.369	1.10(0.87-1.39)	0.431	1.30(0.98-1.73)	0.071
Induced	0.88(0.61-1.25)	0.432	1.07(0.86-1.39)	0.605	0.98(0.70-1.36)	0.889

Abbreviations: VD=vaginal delivery, CS = Caesarean section, CI = confidence interval, OR = odds ratio, ref = reference
Adjusted for maternal and cohort member characteristics, maternal health characteristics, and pregnancy complications as per Model 5
